# Change of primary health care service provider model in Vantaa: the impact on mortality and causes of death among older adults – a register-based follow-up study

**DOI:** 10.1186/s12913-025-12595-y

**Published:** 2025-03-27

**Authors:** Aina Enckell, Hanna-Maria Roitto, Hannu Kautiainen, Mika T. Lehto, Kaisu H. Pitkälä, Timo Kauppila, Merja K. Laine

**Affiliations:** 1 Department of General Practice and Primary Health Care, University of Helsinki and Helsinki University Hospital, Helsinki, Finland; 2 Western Uusimaa Wellbeing Services County, Espoo, Finland; 3 Department of Medicine, Clinicum, University of Helsinki, Helsinki, Finland; 4 Department of Geriatrics, Helsinki University Hospital, Helsinki, Finland; 5 Department of Public Health, Finnish Institute for Health and Welfare, Helsinki, Finland; 6 Folkhälsan Research Centre, Helsinki, Finland; 7 Primary Health Care Unit, Kuopio University Hospital, Kuopio, Finland; 8 Wellbeing Services County of Vantaa and Kerava, Vantaa, Finland

**Keywords:** Access to care, Cause of death, Follow-up study, General practitioner, Named general practitioner, Mortality, Primary health care

## Abstract

**Background:**

Access to primary health care (PHC) has declined in Finland in recent years. To address this, the city of Vantaa, Finland, transitioned from a named general practitioner (GP) model to a restricted-list GP model in 2011 to increase access to named GPs for the most vulnerable population. This study evaluates the impact of this model change on mortality rates and causes of death among older adults.

**Methods:**

This register-based follow-up study was conducted in Vantaa, Finland, using data from the electronic health records. The study included all patient contacts aged 75 and older between 1 September 2004 and 31 August 2018. The primary outcome was the Standardised Mortality Ratio (SMR). We calculated excess deaths and examined cause-specific mortality trends before and after the restricted-list GP model implementation.

**Results:**

During the study period, 32,034 PHC contacts were recorded. The SMR remained stable during the named GP model years but began to decrease during the restricted-list GP model, falling below expected levels from 2016 onward. Excess deaths decreased from 615 in 2004 in the named GP model to -29 by 2018, when the restricted-list GP model was in operation. Leading causes of death were circulatory diseases (41.1%), cancers (20.4%) and neurological conditions (17.8%), with a decrease in circulatory disease deaths and an increase in cancer and dementia-related deaths over time.

**Conclusions:**

The transition to the restricted-list GP model was associated with a lower SMR and fewer excess deaths in older adults. These findings highlight the importance of ongoing assessment of PHC models to ensure they meet evolving healthcare demands.

## Background

Primary health care (PHC) serves as the first point of contact with the healthcare system for all citizens [1]. Effective PHC must operate in an equitable, efficient, and impactful manner to support the health of individuals, the general population, and public health as a whole [2–5]. Accessibility to PHC is a critical factor in improving population health [6], especially for older adults. Better access to PHC reduces mortality [7–12], and the organization of PHC services can substantially influence care access [6].

Multiple efforts have aimed to improve PHC accessibility globally and also in Finland [13–18]. PHC services in Finland are non-profit, primarily funded by taxes, and largely organised through PHC centres accessible to everyone [19]. Despite these efforts, however, access to PHC services in Finland has declined in the last decades [20, 21], during which Finnish health care centers have undergone changes due to societal shifts, financial constraints, and a shortage of experienced primary care professionals. In the 1990s, reduced medical school intakes led to a general practitioner (GP) shortage, worsening working conditions, increasing workload, and affecting care accessibility and continuity [21].

In the 1990s, the city of Vantaa, Finland, implemented PHC based on a named GP model [22], where each GP was responsible for a population in a certain geographic area with approximately 2 000 residents, for whom they served as a named GP. A named GP is a GP assigned to a patient and is in first hand responsible for their care. The named GP takes responsibility for coordinating treatments, ensuring continuity of care, and addressing the patient’s overall health needs. During the named GP model, Vantaa’s GPs experienced high workloads and increased professional demands [23, 24], resulting in a gradual decline in care accessibility as GPs left the health centres due to workload pressures [21, 25, 26]. The number of GPs decreased, and particularly as the number of scheduled appointments dropped [26], patients were often directed to any available GP, and often to urgent scheduled appointments [16]. The economic recession beginning in 2008 further constrained financial resources [27] for health centres. Therefore, in 2011, Vantaa implemented a new restricted-list GP model aiming to ensure access to GPs and continuity of care by assigning older adults aged 75 years or more, individuals with chronic diseases as well as frequent PHC users, to a named GP [18, 25]. GPs prioritised appointments for listed patients, with fewer slots available for non-listed, younger and healthier individuals [28]. The objective was that the new restricted-list GP model would ensure access to non-urgent scheduled appointments and continuity of care for those patients who needed it the most [18].

This study aims to assess the restricted-list GP model by evaluating whether transitioning from the named GP model to the restricted-list GP model affected mortality rates and causes of death among older adults in Vantaa, Finland.

## Methods

### Study design and setting

This register-based follow-up study was conducted in the city of Vantaa, Finland. Vantaa is part of the Helsinki metropolitan area and is Finland’s fourth-largest city. During the study period, Vantaa had a mean population of 9,100 residents aged 75 and older.

Data for the Vantaa PHC centres were obtained from the Graphic Finstar electronic health record system. This electronic health record system enabled data to be analysed at the individual patient and GP levels. Data consisted of all records entered in the electronic health record system during the study period between 1 September 2004 and 31 August 2018 for patients aged 75 years and older. The transition from the named GP model to the restricted-list GP model took place on 1 September 2011.

### Characteristics of the study cohort and type of primary health care contacts

The Charlson comorbidity index (CCI) was calculated to assess each study cohort’s burden of comorbidity both during the named GP model and the restricted-list GP model based on diagnoses retrieved from medical records. The comorbidity registration was done at the point of the beginning of the follow-up period. CCI describes morbidity and predicts mortality [29]. PHC contacts included the following types of contacts: the number of face-to-face scheduled non-urgent appointments, urgent face-to-face office-hours appointments in the health centres and PHC emergency department, paper and phone consultations including prescription renewals, and home visits. CCI and PHC contacts were included to compare cohort characteristics. Data were collected from all seven public PHC centres in Vantaa. Data from the private sector were not available.

### Mortality and death causes

The primary outcome measures in this study were Standardised Mortality Ratios (SMR) for the seven years before and seven years after the transition to the restricted-list GP model. The SMR compares the mortality rate of the study cohort with that of the general population, in this case Finland’s, to what would be expected based on the general population’s mortality rates, taking age and sex into account [30]. Additionally, the number of excess deaths during both models was calculated. The cause of death was defined as the underlying cause of death, coded by the 10th revision of the International Classification of Diseases (ICD-10) classification system [31].

The causes of death were retrieved from death certificates from the Finnish Cause of Death register for deaths registered no later than 31 December 2018. The causes of death were classified as follows: A00-B99 (infectious and parasitic diseases), C00-D48 (neoplasms), D50-D89 (diseases of the blood and blood-forming organs and certain disorders involving the immune mechanism), E00-E90 (endocrine, nutritional and metabolic diseases), F00-F99 (mental and behavioural disorders), G00-G99 (diseases of the nervous system), I00-I99 (diseases of the circulatory system), J00-J99 (diseases of the respiratory system), K00-K93 (diseases of the digestive system), L00-L99 (diseases of the skin and subcutaneous tissue), M00-M99 (diseases of the musculoskeletal system and connective tissue), N00-N99 (diseases of the genitourinary system), Q00-Q99 (congenital malformations, deformations and chromosomal abnormalities), R00-R99 (symptoms, signs and abnormal clinical and laboratory findings, not elsewhere classified), and V01-Y98 (external causes of morbidity and mortality).

### Statistical analysis

Data were expressed as mean and standard deviation (SD) or frequencies with percentages. The ratio between observed and expected numbers of deaths, the SMR and excess mortality (which is the difference between observed and expected mortality rates) were calculated using subject-year methods with 95% confidence intervals (CIs), assuming a Poisson distribution. The expected number of deaths was calculated on the basis of sex-, age-, and calendar-period-specific mortality rates in the Finnish population (Official Statistics of Finland). The expected number was determined by multiplying the number of person-years of observation by the appropriate mortality rate in the general population according to the categories of sex, 1-year age group, and calendar period. Stata V.17.0 (StataCorp) statistical package was used for the analysis.

## Results

During the study period, the total number of contacts was 32,034. Of these contacts, 62% were from women and the mean age of the patients was 78 years. The characteristics of the study cohort, the number of different contacts, and mortality in the study cohort in both models are presented in Table 1. There were no changes in cohort characteristics between the follow-up periods.

**Table 1:** Characteristics of the study cohort, number of deaths and the types of contacts in the named GP model and in the restricted-list GP model

	Named GP-model *n* = 13 336	Restricted-list GP model *n* = 18 698
Timeline	September 2004-August 2011	September 2011-August 2018
Female, n (%)	8 342 (63)	11 385 (61)
Age years, mean (SD)	78.2 (4.6)	78.4 (4.7)
CCI, mean (SD)	1.31 (1.29)	1.13 (1.21)
Contacts, number per 1000 patient years		
Acute	975	1 165
Non-acute	1 962	1 320
Paper and phone consultations including prescription renewal	7 387	7 177
Home visits	79	69
Deaths, n	4364	5531
Person years followed up	104 901	78 201

*GP * General practitioner, *SD * Standard Deviation, *CCI * Charlson Comorbidity Index

**Fig. 1:**
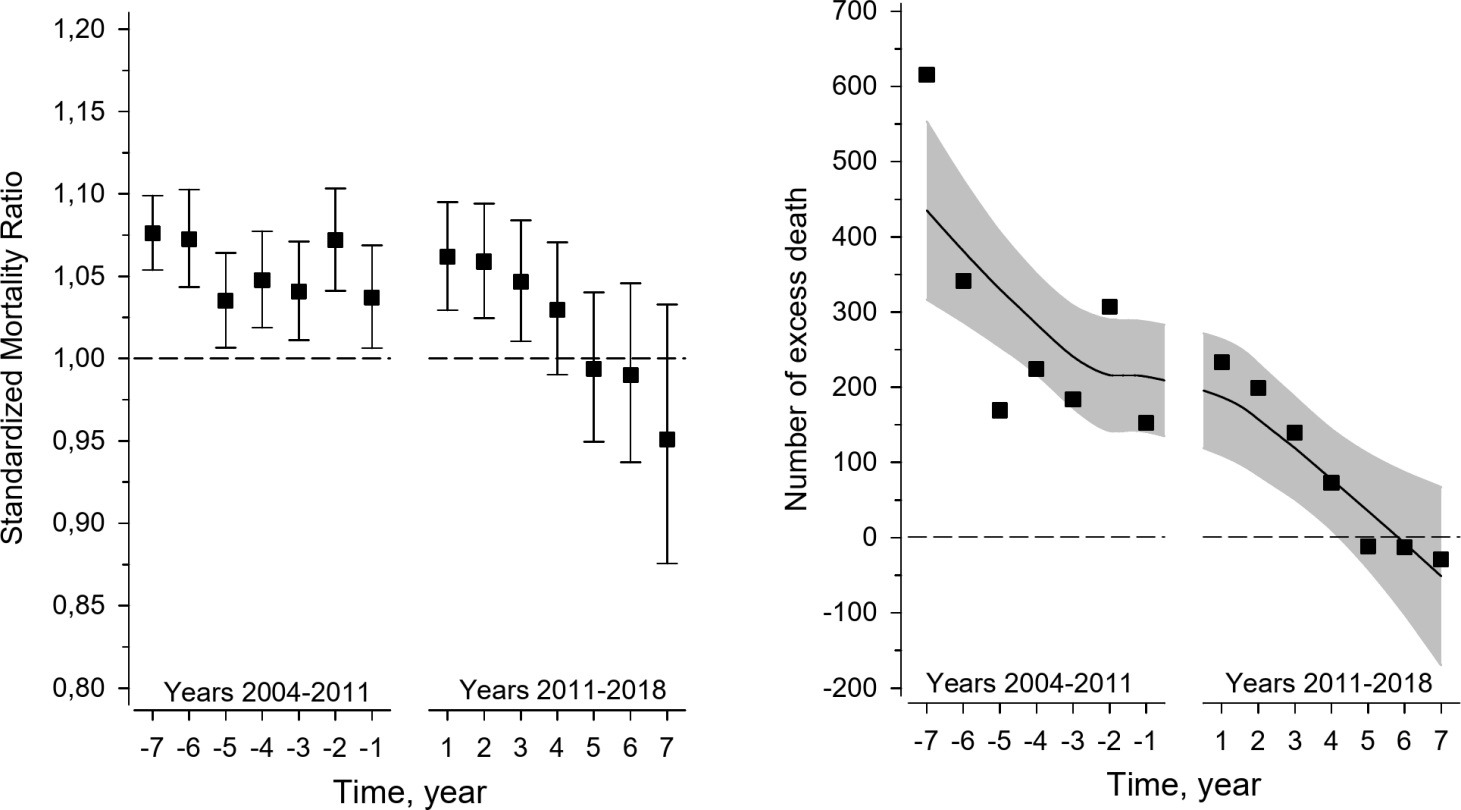
**a** Standardised mortality ratio and **b** Number of excess deaths seven years before and after timepoint of model change. Legends: **a** The figure illustrates the standardised mortality ratio seven years before and after timepoint of model change. The error bars represent the 95% confidence interval. **b** The figure illustrates number of excess deaths seen years before and after timepoint of model change. The line shows the mean, grey areas represent 95% confidence intervals

Figure 1a shows the SMR during the time periods of the named GP model (2004–2011) and restricted-list GP model (2011–2018). SMR was stable during the named GP model and the first years of the restricted-list GP model, starting to decrease in 2015 and was below the expected level from 2016.

Figure 1b shows the number of excess deaths in the named GP model (2004–2011) and the restricted-list GP model (2011–2018). The number of excess deaths decreased steadily during these years from 615 to 73 (2004–2015), and by 2018, it decreased to −29.

The most common cause of death (41.1%) was diseases of the circulatory system, more specifically ischemic heart diseases (21.3%) (ICD-10 codes I21-I25), followed by cancer diagnoses (ICD-10 codes C00-D48), and neurodegenerative diseases including Alzheimer’s disease and other dementia diagnoses (ICD-10 code G30-G32 and F00-F03) (Table 2). The cause of death by heart disease (ICD-10 I00-I99) decreased from 43.7% in the named GP model to 39.0% in the restricted-list GP model, while the cause of death by cancer (ICD-10 C00-D48) increased from 18.9% in the named GP to 21.6% during the restricted-list GP model. The underlying cause of death by dementia diagnoses (ICD-10 codes F00-F03 and G30-G32) increased from 19.7% in the named GP model to 23.6% during the restrict-list GP model.

**Table 2:** Description of cause of death (%) divided into ICD-10 diagnostic codes in the named GP model and the restricted-list GP-model

Cause of death, ICD-10 codes	Named GP model (%)	Restricted-list GP model (%)	Total (%)
A00-B99 infectious and parasitic diseases	0.99	0.25	0.58
C00-D48 neoplasms	18.88	21.64	20.42
D50-D89 diseases of the blood and blood-forming organs and certain disorders involving the immune mechanism	0.11	0.09	0.10
E00-E90 endocrine, nutritional and metabolic diseases	0,78	0.80	0.79
F00-F99 mental and behavioural disorders	7.97	5.88	6.80
G00-G99 diseases of the nervous system	13.91	20.83	17.78
I00-I99 diseases of the circulatory system	43.72	39.02	41.09
J00-J99 diseases of the respiratory system	5.89	4.65	5.15
K00-K93 diseases of the digestive system	2.75	2.69	2.72
L00-L99 diseases of the skin and subcutaneous tissue	0.09	0.00	0.04
M00-M99 diseases of the musculoskeletal system and connective tissue	0.60	0.36	0.46
N00-N99 diseases of the genitourinary system	1.24	0.63	0.90
Q00-Q99 congenital malformations, deformations and chromosomal abnormalities	0.00	0.07	0.04
R00-R99 symptoms, signs and abnormal clinical and laboratory findings, not elsewhere classified	0.09	0.29	0.20
V01-Y98 external causes of morbidity and mortality	2.89	2.68	2.77
Unknown	0.18	0.13	0.15

*GP * General practitioner, *ICD-10 * International Classification of Diseases, 10^th^ version

## Discussion

In this study we found that SMR and excess number of deaths decreased after changing the service provider model to the restricted-list GP model. During the named GP model period, the SMR remained consistently higher in the study cohort than in the general Finnish population. At the same time, the number of excess deaths steadily decreased throughout the study period, reaching the same level and even below that of the general population by 2015–2018. Cardiovascular reasons for death were the most common and tended to decrease while neurologic disorders and cancers became more common.

In numerous studies it has been stated that continuity of care in PHC reduces mortality [32–35]. The aim of the restricted-list GP model was to enhance access to and continuity of care for the ill and the elderly, compared to the named GP model. In both models, the study cohort was assigned to a named GP. We can speculate that continuity of care may have been realised better in the restricted-list GP model, since the patients didn’t have to compete for appointments with the less ill population, as the listed patients had access to allocated appointments. The result is also in line with findings that the numbers of banal diagnoses (such as upper respiratory infections) treated by GPs in Vantaa have decreased during the years of our study [36–38]. Thus, we can speculate that the GPs used their time to treat more severe diseases after the model change.

The shift from the named GP model to the restricted-list GP model was associated with a decrease in SMR and number of excess deaths. As these parameters are definite indicators of patient safety [15, 39] it seems that patient safety was not endangered by the change to the restricted-list GP model. However, mortality and related outcomes are influenced by various factors, and they are rarely affected by PCH actions or system changes alone. For instance, reducing PHC patients’ access to physicians in emergency services [39, 40] or decreasing the number of available PHC emergency departments [41] did not alter mortality rates. Similarly, transitioning to a pay-for-performance system for treating serious and under-treated illnesses in PHC had no impact on mortality [15]. Furthermore, an earlier study found that decrease in office-hour appointments with GPs did not affect mortality rates in older adults [36].

The cause of death diagnoses shifted before and after the change to the restricted-list GP model. Circulatory diseases tended to decrease as a cause of death. This is in line with recent findings in developed countries [42]. It is possible that circulatory disease treatment has improved throughout the years, which may explain this. The increase in the share of cancer and dementia related deaths is also in line with current literature [43–45]. The population is ageing, which may explain these results. The amount of 75 year and older citizens in Vantaa increased during the study period [36].

One of the strengths of this study was that Finnish public PHC provides services to the entire population. The study uses comprehensive, real-world data from the Vantaa PHC electronic health record system, making findings applicable to practical healthcare settings. The large dataset over a 14-year period allows for a strong analysis of trends. By targeting patients aged 75 years and older, the study reports the needs of a vulnerable patient group that frequently requires continuous and accessible care.

However, the study also has limitations. Since the data is limited to Vantaa, they do not fully represent the entire Finnish population, given Vantaa’s own demographic and socioeconomic characteristics. In our data women used the PCH centres more frequently, which is in line with the previous literature [46, 47]. The number of pensioners was smaller in Vantaa than in the whole country (18% vs. 26% in 2017). The proportion of foreign citizens in Vantaa was higher compared to that of the whole country (12% vs. 5%) in 2018 (https://stat.fi/tup/alue/kuntienavainluvut.html#?active1=KU092&active2=SSS&year=2023).

Furthermore, we only had access to public PHC contacts and lacked information on the studied patient group’s use of the private sector during these years, which could have affected mortality. As stated earlier, continuity of care affects mortality [32–34]. However, we were not able to measure the continuity of care during these years in this population. Variations in population mortality are predicted mainly by population characteristics such as deprivation [12, 48, 49] and we are missing data on the patient’s life circumstances and whether they changed during the study period. Additionally, we are unaware of whether the quality of care changed. We do not know if the named GPs work strain and work circumstances changed during the restricted-list GP model, although this was one of the aims of the model change. This is to be studied in further research.

Although our data were large and the follow-up period was long, the number of deaths remained small. Therefore, a more detailed analysis of individual causes of death was not warranted. This study is a follow-up study using only SMR (whole Finland) as a control group; therefore, true causal relationships between the PHC service provider model and mortality cannot be assessed. Given the constantly shifting environment of PHC, it is challenging in a long-term follow-up study like this one to fully account for secular trends and events that may influence the results. For example, in Vantaa during these years there were major changes in emergency services which became outsourced to secondary health care in the end of 2014 [50]. Additionally, in 2009–2010, the Swine flu pandemic occurred which reduced access to PHC services [38].

## Conclusions

Before 2011 the SMR was steady and after transitioning to the restricted-list model, SMR started to decrease. The decrease in the SMR indicates that patient safety was maintained after the model change. While cardiovascular diseases declined as causes of death, neurodegenerative diseases and cancers became more prevalent. Continuous assessment of healthcare system changes is essential, and further research is warranted to explore the broader implications of healthcare access on population health.

## Data Availability

Data are not available. The data used in this study are not publicly available due to privacy and ethical restrictions. Access to the anonymized data was granted solely to the research team, as per the ethical board’s guidelines.

## Abbreviations

CCI: Charlson comorbidity index
CI: Confidence interval
GP: General practitioner
ICD-10: International Classification of Diseases, 10^th^ version
SD: Standard deviation
SMR: Standardised mortality rate
PHC: Primary health care
